# Endoscopic intervention versus radical nephroureterectomy for the management of localized upper urinary tract urothelial carcinoma: a systematic review and meta-analysis of comparative studies

**DOI:** 10.1007/s00345-024-05032-y

**Published:** 2024-05-14

**Authors:** Carlo Giulioni, Carlo Brocca, Pietro Tramanzoli, Silvia Stramucci, Matteo Mantovan, Leonard Perpepaj, Andrea Cicconofri, Vineet Gauhar, Axel Stuart Merseburger, Andrea Benedetto Galosi, Daniele Castellani

**Affiliations:** 1 Department of Urology, Urology Unit, Azienda Ospedaliero-Universitaria delle Marche, Polytechnic University of Marche, 71 Conca Street, 60126 Ancona, Italy; 2 Department of Urology, Ng Teng Fong General Hospital, Singapore, Singapore; 3 Department of Urology, University Lübeck, University Hospital Schleswig-Holstein, Lübeck, Germany

**Keywords:** Localized upper urinary tract urothelial carcinoma, Radical nephroureterectomy, Nephron-sparing surgery, Endoscopic treatment, Survival

## Abstract

**Objective:**

Localized Upper Urinary Tract Urothelial Carcinoma (UTUC) is an uncommon cancer typically detected at an advanced stage. Currently, radical nephroureterectomy (RNU) with bladder cuff excision is the standard treatment for high-risk UTUC. This meta-analysis aims to evaluate the 5-year overall and cancer-specific survival and bladder recurrence rates in studies comparing endoscopic kidney-sparing surgeries (E-KSS) with RNU in localized UTUC.

**Evidence acquisition:**

We performed a literature search on 20th April 2023 through PubMed, Web of Science, and Scopus. The PICOS model was used for study inclusion: P: adult patients with localized UTUC; I: E-KSS. C: RNU; O: primary: overall survival (OS); secondary: cancer-specific survival (CSS), bladder recurrence rate, and metastasis-free survival (MFS). S: retrospective, prospective, and randomized studies.

**Evidence synthesis:**

Overall, 11 studies involving 2284 patients were eligible for this meta-analysis, 737 in the E-KSS group and 1547 in the RNU group. E-KSS showed a similar overall 5-year OS between E-KSS and RNU, and for low-grade tumors, while 5-year OS favored RNU for high-grade tumors (RR 1.84, 95% CI 1.26–2.69, p = 0.002). No difference emerged for 5-year CSS between the two groups, even when the results were stratified for low- and high grade tumors. Bladder recurrence rate and 5-year MFS were also similar between the two groups.

**Conclusions:**

Our review showed that E-KSS is a viable option for patients with localized UTUC with non-inferior oncological outcomes as compared with RNU, except for 5-year OS in high-grade tumors which favoured RNU.

**Supplementary Information:**

The online version contains supplementary material available at 10.1007/s00345-024-05032-y.

## Introduction

Upper Urinary Tract Urothelial Carcinoma (UTUC) is an uncommon tumor that occurs in fewer than 2 out of every 100,000 people and comprises around 5–10% of all urothelial tumors [1]. UTUC is often diagnosed at an advanced stage, accounting for approximately 60% of cases, with a 5-year overall survival of 57% [2, 3]. Unlike bladder cancer (BC), preoperative histology and imaging for staging are often inaccurate, with a high recurrence rate and risk of progression due to tumor biology [4]. The recommended treatment for high-risk UTUC is radical nephroureterectomy (RNU) with bladder cuff excision [5], although RNU can be associated with long-term renal impairment. Solitary kidney condition can be debilitating as UTUC is more common in the elderly, who often have comorbidities already impacting their renal function, such as hypertension or diabetes mellitus, with a higher risk of developing chronic kidney disease with subsequent increased overall mortality [6].

Historically, kidney-sparing surgeries (KSS), including endoscopic ablation/resection and segmental ureterectomy, were offered only in selected cases such as those with a solitary kidney, chronic kidney disease, or bilateral disease [7]. KSS was primarily recommended for patients with low-grade tumors and guaranteed satisfactory oncological radicality [8]. According to the latest systematic review and meta-analysis, there was no significant difference in overall survival and cancer-specific survival between patients who underwent RNU and those who received endoscopic KSS (E-KSS) [9]. Due to technological advances and surgical experience, endoscopic treatment is nowadays offered to a larger number of UTUC patients. Indeed, the most recent AUA guidelines suggest that a tumor ablation is a valid option for patients with low-risk favorable UTUC. Furthermore, it is weakly recommended even for selected patients with high-risk favorable diseases who have low-volume tumors or cannot undergo RNU [10]. Similarly, the updated EAU guidelines suggest offering KSS as a primary treatment in patients with low-risk UTUC and two functional kidneys [5].

This study aimed to perform a systematic review and meta-analysis to compare the overall survival (OS) of patients with localized UTUC comparing E-KSS versus RNU. Secondary outcomes were cancer-specific survival (CSS), bladder recurrence rate and metastasis-free survival (MFS).

## Evidence acquisition

### Literature search

This systematic review was performed according to the 2020 Preferred Reporting Items for Systematic Reviews and Meta-Analyses (PRISMA) statement.

Literature search was performed on 20th April 2023 using PubMed, EMBASE, and Scopus with no date restriction. The following terms and Boolean operators were used: (Conservative treatment OR endoscopic treatment OR nephron-sparing surgery OR laser surgery) AND (Upper Urinary Tract OR collecting system OR pelvis OR ureter) AND (Urothelium cancer OR Urothelial Carcinoma OR UTUC). The review protocol was registered in PROSPERO with the registration number (CRD42023423778).

### Selection criteria

The PICOS (Patient, Intervention, Comparison, Outcome, Study type) model was used to frame and answer the clinical question: P: adult patients with localized UTUC; I: endoscopic conservative treatments (i.e. ureteroscopy or percutaneous ablation/resection). C: nephroureterectomy; O: primary: OS; secondary: CSS, bladder recurrence rate, MFS. S: retrospective, prospective, and randomized studies.

### Study screening and selection

Studies were accepted based on PICOS eligibility criteria. Only English papers were accepted. Pediatric, preclinical, and animal studies were excluded. Case reports, reviews, letters to the editor, and meeting abstracts were excluded. Studies with no data for meta-analysis and with less than 20 patients in the conservative group were also excluded. All retrieved studies were screened by two independent authors through Covidence systematic review software (Veritas Health Innovation, Melbourne, Australia). A third author solved discrepancies. The full text of the screened papers was selected if found pertinent to the aim of this review.

### Statistical analysis

Overall and cancer-specific survival, and recurrence rates were pooled using the Cochran-Mantel–Haenszel Method with the random effect model and reported as risk ratio (RR), 95% confidence interval (CI), and p-value. Subgroup analyses were performed for low-grade and high-grade tumors. Study heterogeneity was assessed utilizing the I^2^ value. Considerable heterogeneity was defined as an I^2^ value between 75 and 100%. Statistical significance was set at two tails p-value < 0.05 and 95% CI. Meta-analysis was performed using the computer program Review Manager (RevMan) version 5.4 (the Cochrane Collaboration, 2020). The quality assessment of the included studies was performed using the Cochrane Risk of Bias tool, using ROBINS-I for retrospective and prospective non randomized studies [11].

## Evidence synthesis

### Literature screening

Literature search retrieved 2614 papers. 489 duplicates were automatically excluded. 2125 papers were screened against title and abstract and 1851 papers were further excluded because were unrelated to the aim of the present review. The remaining 274 full-text papers were evaluated for eligibility and 263 studies were excluded. Finally, 11 papers were accepted and included [12–22]. Supplementary Fig. 1 shows the flow diagram of the literature search.

### Study characteristics

Table 1 shows the study characteristics. All included studies were retrospective. Overall, there were 2284 patients included in 11 studies, 737 patients in the E-KSS and 1547 patients in the RNU group.

**Table 1:** Studies comparing Endoscopic kidney-sparing surgery (E-KSS) and radical nephroureterectomy (RNU) in patients with upper urinary tract urothelial carcinoma (UTUC)

First Author (Year)	Country	E-KSS, n	Age, years	Grade (HG/G3), n (%)	Follow-up, months	Overall Survival, %	Cancer-specific survival, %	Bladder recurrence rate, %	RNU, n	Age, years	Grade (HG/G3), n (%)	Follow-up, months	Overall Survival, %	Cancer-specific survival, %	Bladder recurrence rate, %	Conclusions
Chen (2021) [12]	Taiwan	84	68.8	43 (51.19%)	33.5	5-y: 85%	5-y: 89%	35	272	67.6 ± 10.6	84 (30.88%)	42,0	5-y: 75%	5-y: 90%	39%	E-KSS achieved comparable OS, CSS and bladder recurrence free survival as RNU in high grade UTUC
Cutress (2013) [13]	UK	59	69.4	6 (10%)	50	5-y: 64.1% 10-y: 31.9%	5–y: 85.6% 10–y: 68.3%	33.2	70	78.1	16 (23%)	49,5	5-y: 74.8% 10-y: 52.0%	5-y: 92.1% 10-y: 65.0%	22,7%	For LG UTUC, E-KSS had similar 5- year CSS to RNU. For HG disease, E-KSS showed inferior oncological outcomes
Fajkovic (2013) [14]	Austria	20	71.9	3 (15%)	60	5-y: 45%	5–y: 67%	15	178	68.9 ± 10.8	44 (25%)	60	5-y: 76%	5-y: 91%	36%	E-KSS showed similar rate and CSS, while OS was lower than RNU
Gadzinski (2010) [15]	USA	34	-	8 (23.5%)	76.9	5-y: 75% 1) LG 74.8% 2) HG: 25.0%	5–y: 1) LG: 100% 2) HG: 85.7%	–	62	–	34 (55%)	76,9	5-y: 72% 1) LG: 71.8% 2) HG: 47.8%	5-y: 1) LG: 89.2% 2) HG: 72.4%	–	E-KSS and RNU showed similar outcomes, although the latter should be preferred for HG tumors
Hoffman (2014) [16]	Israel	25	64	0 (0)	26	-	5–y: 100%	44	22	76	–	57	–	–	–	E-KSS for LG UTUC is associated with similar CSS, but it has a higher rate than RNU
Lee (1999) [17]	USA	49	-	G1:20 (40.8%) G2:16 (32.3%) G3:13 (26.5%)	46.6	-	–	–	57	–	G1: 6 (10.5%) G2: 30 (52.6%); G3: 21 (36.8%)	46,6	–	–	–	Tumor grade was the worst prognostic factor. CSS between E-KSS and RNU was similar
Lucas (2008) [18]	USA	39	68	12 (31%)	33	5-y: 61.7%) 1) LG: 75.4% 2) HG: 45%	–	–	90	65	56 (73%)	46	5-y: 72.1% 1) LG: 66.4% 2) HG:71.5%	–	–	5-year CSS for low grade tumors was similar between E-KSS and RNU
Raymundo (2011) [19]	USA	21	68.8	6 (30%)	17.9	-	–	10	99	72.9	46 (49%)	17,9	–	–	16%	Patients treated with E-KSS had a higher BR rate
Seisen (2016) [20]	France	42	69	5 (11.9%)	29.1	5-y: 74.4%	5-y: 83.3%	41.1	128	68 (59–75)	87 (68%)	30,6	5-y: 73.5%	5–y: 87.4%	53,3%	E-KSS offered a higher OS and similar CSS compared to RNU
Shen (2022) [21]	Taiwan	23	66.0	–	33.6	5-y: 94.5%	–	–	42	69.3	−( 85.7%)	33,6	5-y: 94.6%	–	–	E-KSS showed similar oncological outcomes than RNU, with a greater renal function preservation
Upfill-Brown (2019) [22]	USA	323	< 65 y:552 65–79 y: 1379 80 + : 1039	–	–	5-y: 69.3%	–	–	527	< 65 y: 3599 65–79 y: 6953 > 80 y: 3261	–	–	5-y: 75.2%	–	–	RNU was associated with higher 5-years OS than E-KSS

*HG* high-grade; *LG* low-grade; *OS* overall survival; *CSS* cancer-specific survival; *BR* bladder recurrence; *y* year

There were 4 ureteroscopy studies [15, 17, 19, 21] and 1 percutaneous study [20]. Four studies employed both ureteroscopic and percutaneous approaches [13, 14, 18, 22], while the remaining ones did not specify which type of conservative treatment was applied [12, 16]. Five studies did not specify the energy source for conservative treatment [12, 15, 17, 18, 21], 2 studies used electrocautery [20, 22], one study used laser energy, and both electrocautery and laser energies were used in the remaining ones [14, 16, 19].

### Risk of bias assessment

Supplementary Fig. 2 shows the details of the quality assessment for the included studies. Overall, 4 studies showed serious risks of bias, and the remaining ones had a moderate risk of bias. The most common reason for bias was bias due to confounding, followed by bias due to the selection of participants.

### Primary outcome

#### 5-year overall survival

**Fig. 1:**
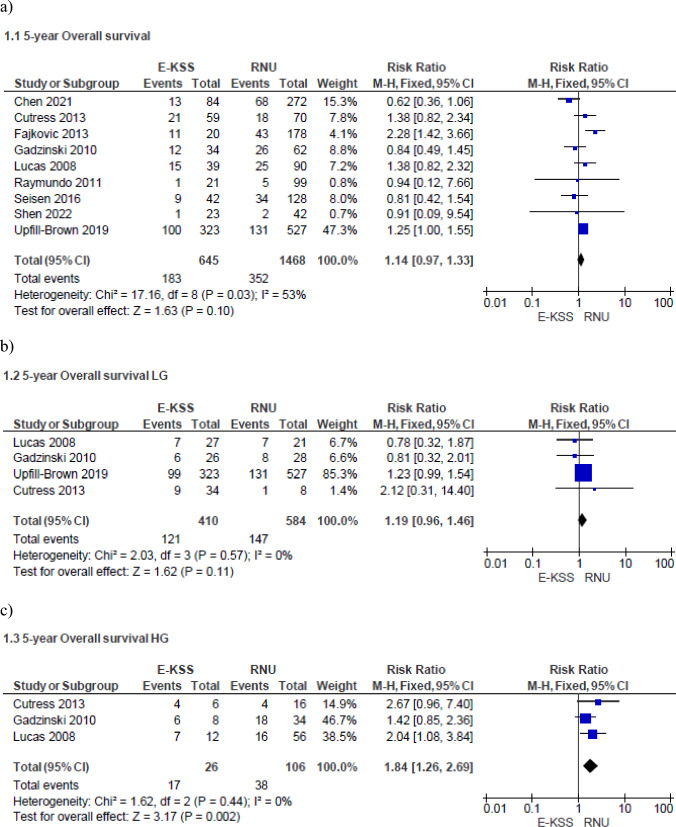
Forrest plots of 5-year Overall Survival of studies comparing Endoscopic kidney-sparing surgery (E-KSS) versus Radical nephroureterectomy (RNU). Analysis of: overall tumors (**a**); low-grade tumors (**b**); high-grade tumors (**c**)

Meta-analysis from nine studies (645 cases in E-KSS and 1468 cases in RNU) showed that the 5-year OS is similar between E-KSS and RNU groups (RR 1.14, 95% CI 0.97–1.33, p = 0.10) (Fig. 1a). Study heterogeneity was moderate (I^2^ 53%).

When stratified for tumor grade, meta-analysis from 4 studies (410 cases in E-KSS and 584 cases in RNU) showed that there was no difference between the E-KSS and RNU group for low-grade tumors (RR 1.19, 95% CI 0.96–1.46, p = 0.11) (Fig. 1b). Study heterogeneity was not important (I^2^ = 0%).

Meta-analysis from 3 studies (26 cases in E-KSS and 106 cases in RNU) showed that 5-year OS for high-grade tumors (Fig. 1c) significantly favors RNU (RR 1.84, 95% CI 1.26–2.69, p = 0.002). Study heterogeneity was not important (I^2^ = 0%).

### Secondary outcomes

#### 5-year cancer-specific survival

**Fig. 2:**
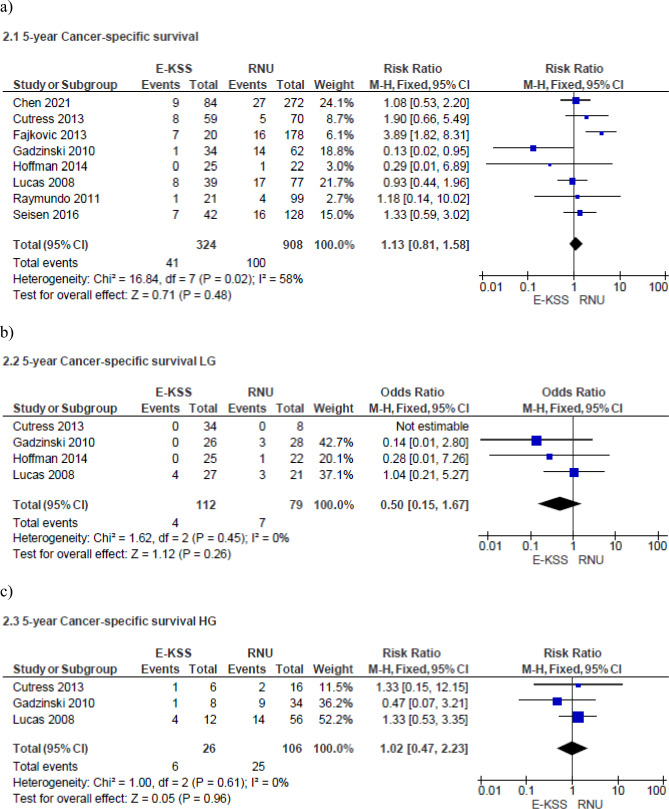
Forrest plots of 5-year cancer specific Survival of studies comparing Endoscopic kidney-sparing surgery (E-KSS) versus Radical nephroureterectomy (RNU). Analysis of: overall tumors (**a**); low-grade tumors (**b**); high-grade tumors (**c**)

Meta-analysis from eight studies (324 cases in E-KSS and 908 cases in RNU) showed no significant difference in 5-year CSS between E-KSS and RNU groups (RR 1.13, 95% CI 0.81–1.58, p = 0.48) Study heterogeneity was moderate (I^2^ 58%) (Fig. 2a).

When stratified for tumor grade, meta-analysis from 4 studies (112 cases in E-KSS and 79 cases in RNU) showed no difference in 5-year cancer-specific survival for low-grade UTUC between the two groups (RR 0.50, 95% CI 0.15–1.67, p = 0.26) (Fig. 2b). Study heterogeneity was not important (I^2^ = 0%). Even for high-grade tumors, meta-analysis from three studies (26 cases in E-KSS and 106 cases in RNU) showed similar results in 5-year CSS between E-KSS and RNU groups (RR 1.02, 95% CI 0.47–2.23, p = 0.96) (Fig. 2c). Study heterogeneity was not important (I^2^ 0%).

#### Bladder recurrence rate

**Fig. 3:**
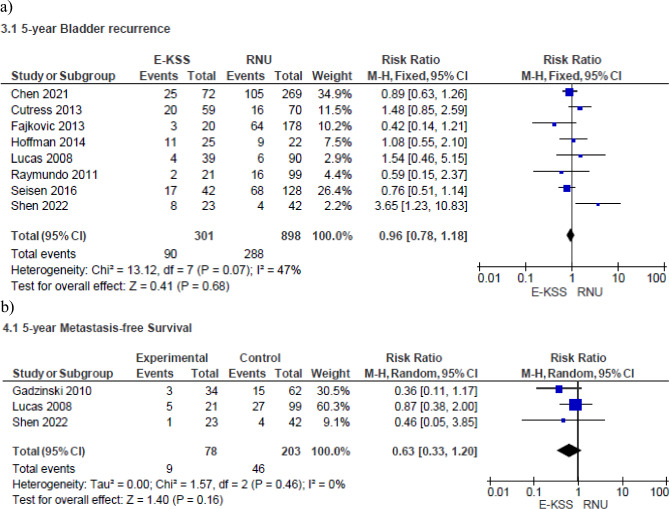
Forrest plot of the Bladder recurrence rate (**a**) and 5-year Metastasis-free Survival (**b**) of studies comparing Endoscopic kidney-sparing surgery (E-KSS) and Radical nephroureterectomy (RNU)

Meta-analysis from eight studies (301 cases in E-KSS and 898 cases in RNU) showed no significant difference in terms of bladder recurrence between the two groups (RR 0.96, 95% CI 0.78–1.18, p = 0.68) (Fig. 3a). Study heterogeneity was moderate (I^2^ = 47%).

#### 5-year metastasis-free survival

Meta-analysis from three studies (78 cases in E-KSS and 203 cases in RNU) showed no significant difference in terms of 5-year MFS between the two groups (RR 0.63, 95% CI 0.33–1.20, p = 0.16) (Fig. 3b). Study heterogeneity was not important (I^2^ = 0%).

## Discussion

This systematic review and meta-analysis demonstrate that E-KSS exhibits non-inferiority to RNU in terms of 5-year overall OS and low-grade UTUC between E-KSS and RNU, 5-year CSS rates for overall, low- and high-grade tumor patients, bladder recurrence rate, and 5-year MFS. However, there was a superior 5-year OS rate for the high-grade localized UTUC patients in the RNU group.

UTUC is recognized to have a relatively increased risk for progression compared to BC, primarily attributable to its distinct embryonic origins, leading to substantial variations in phenotypical and genotypical characteristics [23]. Despite the two tumors share similar risk factors and originate from the urothelium, the location of the urothelial neoplasia in the upper and lower tracts exhibits distinct differences in their behavior [24].

These dissimilarities bear significant implications for their management. Recent advancements in molecular research have elucidated distinctive gene and protein expression patterns between BC and UTUC, thus underscoring their divergent genetic and epigenetic profiles. Additionally, UTUC predominantly manifests as luminal papillary structures and T-cell depletion depicting prominent immunopathological features [25]. UTUC is diagnosed as invasive upon initial detection in 60% of cases, and 15–25% of them are found to be associated with bladder tumors, while approximately 7% exhibit distant metastasis at the time of diagnosis. It is important to highlight that the 5-year CSS rate for UTUC patients is estimated to range between 50 and 80% [26]. Despite the advancements in surgical and imaging technology, accurately identifying low-grade tumors is still difficult due to the potential for underestimation of upper tract invasion associated with endoscopic biopsy procedures [27]. Therefore, the accurate detection and characterization of these tumors pose significant challenges.

In our analysis, E-KSS showed a similar 5-year CSS as compared to RNU. Various recommendations have emerged from the literature regarding the RNU setting, ensuring adequate oncologic outcomes. In a systematic review including 42 studies with over 7,000 patients, open RNU remains the choice treatment since laparoscopic RNU with bladder cuff excision was associated with worse CSS and OS [28]. The management in high-volume centers was also associated with better short-term outcomes (30- and 90-day mortality) and overall long-term survival [29]. Overall, E-KSS has been consistently shown to be similar in achieving oncological outcomes compared to RNU. These findings challenge the notion that comprehensive removal of the primary tumor along with all detectable cancerous tissue and surrounding tissue leads to substantial improvements in patient outcomes and long-term survival in localized UTUC, despite the tumor grade.

Furthermore, RNU is associated with several drawbacks. In a comprehensive study involving a large cohort of 1300 patients who underwent RNU across 14 different centers, the authors found that more than 32% of patients experienced complications, with a mortality rate of 10% [30]. Moreover, it is well-known that nephrectomy is associated with a reduced life expectancy and increased risk of end-stage renal disease due to reduced GFR, proteinuria, and higher blood pressure that arise after the procedure. The decrease in renal function following nephrectomy is also associated with a higher risk of cardiovascular disease development and may predict cardiovascular morbidity and fatal events. The presence of CKD was associated with lower 2-year survival in patients with coronary artery disease (77% vs 87%), acute myocardial infarction (69% vs 82%), heart failure (65% vs 76%), atrial fibrillation (70% vs 83%), and cerebrovascular accident/transient ischemic attack (73% vs 83%), when compared to patients with normal serum creatinine levels [31]. The aforementioned factors may contribute to understanding our findings on similar 5-year OS rate between patients who had a conservative and radical treatment, implying that E-KSS can be a viable option in selected UTUC patients, such as elderly patients suffering from CKD.

RNU demonstrates a significantly superior 5-year OS compared to RNU-only patients with high-grade UTUC. However, according to the European Association of Urology guidelines, tumor grade is just one of the factors contributing to risk stratification [5]. As demonstrated by Upfill-Brown et al., through the inverse probability of treatment-weighted analysis, when considering tumors < 1 cm instead of 2 cm, the significance between the two techniques diminishes [16]. Therefore, a comprehensive evaluation of all risk factors is necessary for assessing appropriate therapeutic management. Feasibility of a complete local ablation is also mandatory before decision-making.

In a previous meta-analysis conducted by Yakoubi et al., a higher incidence of local recurrence was observed in patients undergoing conservative therapy compared to RNU [9]. This finding aligns with the tendency towards the multifocality of UTUC and the hypothesis of cells spreading during conservative treatments. However, our findings indicate that bladder recurrence and distant metastasis rates did not differ between the two groups, pointing out another favorable point in performing E-KSS.

Ureteral recurrence is a pitfall of conservative UTUC treatments. According to Cutress et al. 77% of patients treated with E-KSS were found to have local recurrence over time, but most of these lesions were small and treated through further endoscopic procedures [13]. Therefore, additional ureteroscopy of the ipsilateral upper urinary tract can be considered a safe and feasible procedure during patient follow-up, and lifelong monitoring is mandatory to avoid large recurrent tumors detected upon symptoms.

The tumor grade plays a pivotal role in UTUC management as it provides vital prognostic information that aids in risk stratification. In a comprehensive model designed to predict non-organ confined UTUC, tumor grade emerged as an independent and highly significant factor in determining the likelihood of muscle-invasive disease [32]. Interestingly, the correlation between tumor grade and survival and recurrence rates appears more important than the impact of treatment choice, whether conservative or radical [33]. According to a study involving UTUC patients who underwent endoscopic surgery within 30 years, the authors found that tumor grade remained a key factor in predicting disease recurrence [34]. However, its impact on OS was not deemed to be significant. Conversely, Grasso et al. showed that patients with high- and low-grade disease treated with radical treatment had a 10-year CSS rate of 38% and 89%, respectively [35]. Moreover, the initial tumor grade had a notable influence on both OS (HR = 3.78 95% CI 2.11 – 6.80, p < 0.001) and CSS (HR = 7.14 95% CI 3.25 – 15.7, p < 0.0001) in the multivariate analysis.

Conversely, our analysis found that 5-year CSS in high-grade tumor patients treated with E-KSS was not inferior to the RNU group. Therefore, urologists might offer patients with localized high-grade tumors the appropriate treatment evaluating the similar outcomes related to disease and better mid and long-term OS in a conservative approach. Similarly, our findings point out that E-KSS is a valid option for patients with low-grade tumors. Therefore, a conservative approach with scheduled follow-up visits, imaging and endoscopy can be safely offered even in those patients with a normal contralateral kidney and low-grade tumor when resection/ablation is technically feasible [36]. However, close monitoring with ureteroscopy and appropriate imaging is associated with a burden on patients. Therefore, it is also crucial to consider this when engaging in shared decision-making with the patient regarding the treatment to be pursued. Probably, patients unwilling/unsuitable for close and strict follow-up are not good candidates for UTUC conservative management.

Bladder recurrence in UTUC patients commonly occurs, with around 50% of patients experiencing at least an episode during their follow-up [37]. Bladder recurrence risk is associated with various factors, including tumor characteristics (such as tumor grade, multifocality, and size) and the surgical approach [5]. Furthermore, the observed hazard can also be attributed to variations in techniques, energy application during the endoscopic surgery, laparoscopic technique, bladder cuff excision, and positive surgical margins in RNU. In a comprehensive review conducted by Lucca et al., bladder recurrence was found to be independently influenced by both patient-related and tumor-related factors [38]. Notably, the rates of bladder recurrence were similar in the endoscopic-conservative approach group compared to the RNU group. Consequently, adopting an endoscopic-conservative approach does not appear to result in a higher bladder recurrence rate when compared to RNU. Our analysis, which included 1199 patients, revealed the same and the fear of bladder recurrence should not be a deferral reason against the use of E-KSS since bladder recurrence mostly relies upon patient-related factors.

### Strengths and limitations

We conducted a comprehensive meta-analysis, incorporating studies that collectively provide a wealth of evidence, thereby determining situations in which upfront E-KSS may be recommended. We reported that current evidence supports E-KSS in all UTUC cases in 5-year CSS, and bladder recurrence rate, provided they can be adequately staged preoperatively using available clinical, pathological, genetic, and molecular markers. This staging aspect still presents the highest challenge in UTUC management.

Our meta-analysis reveals that 5-year OS for high-grade tumors favors RNU. However, the number of patients considered appears limited, especially in the E-KSS group (only 26 cases). Therefore, our results should be interpreted with caution, and further studies are needed to draw definitive conclusions. Moreover, perhaps the next focus should shift toward better understanding the underlying biological behavior of UTUC, enabling personalized treatments. One potential possibility is to consider immunological and clinical profiling to guide treatment, as has been advocated for other cancers.

However, we believe that further research in this area would be relevant, particularly with the introduction and utilization of newer lasers [39], percutaneous or ureteroscopic chemotherapeutic gels [40], and technologies such as Optical Coherence Tomography that are showing promising initial results in this field [41].

## Conclusion

Our findings demonstrate the oncological safety of E-KSS in the management of localized UTUC with a non-inferior 5-year OS and 5-year CSS in all tumor grades after E-KSS compared with RNU. However, the differences in survival rates are not significant for high-grade tumors, as the 5-year OS favors RNU. Nevertheless, E-KSS has demonstrated similar bladder recurrence rate and MFS to RNU. Therefore, E-KSS can be considered a viable treatment option, even in patients with normal contralateral kidneys. Nevertheless, a strict long-term follow-up and surveillance ureteroscopy are crucial for the timely detection and treatment of local recurrences.

## Supplementary Information

Below is the link to the electronic supplementary material.

Supplementary file1 (DOCX 323 KB)

Supplementary file2 (DOCX 57 KB)

## Data Availability

The datasets used and analyzed during this study are available from the corresponding author upon reasonable request.
