# Supplementary material for: Endoscopic intervention versus radical nephroureterectomy for the management of localized upper urinary tract urothelial carcinoma: a systematic review and meta-analysis of comparative studies
Source: World J Urol. 2024 May 14;42(1):318. doi: 10.1007/s00345-024-05032-y (PMC11093876; doi:10.1007/s00345-024-05032-y)
Supplement: Supplementary file 1 — Supplementary file1 (DOCX 323 KB) [file 345_2024_5032_MOESM1_ESM.docx]

**Supplementary Figure 2.** *Risk of bias of the included study (ROBINS-I):* ***a)*** *review authors’ judgments about each risk of bias item presented as percentages across all included studies; b****)*** *review authors’ judgments about each risk of bias item for each included study.*

***a)***

**
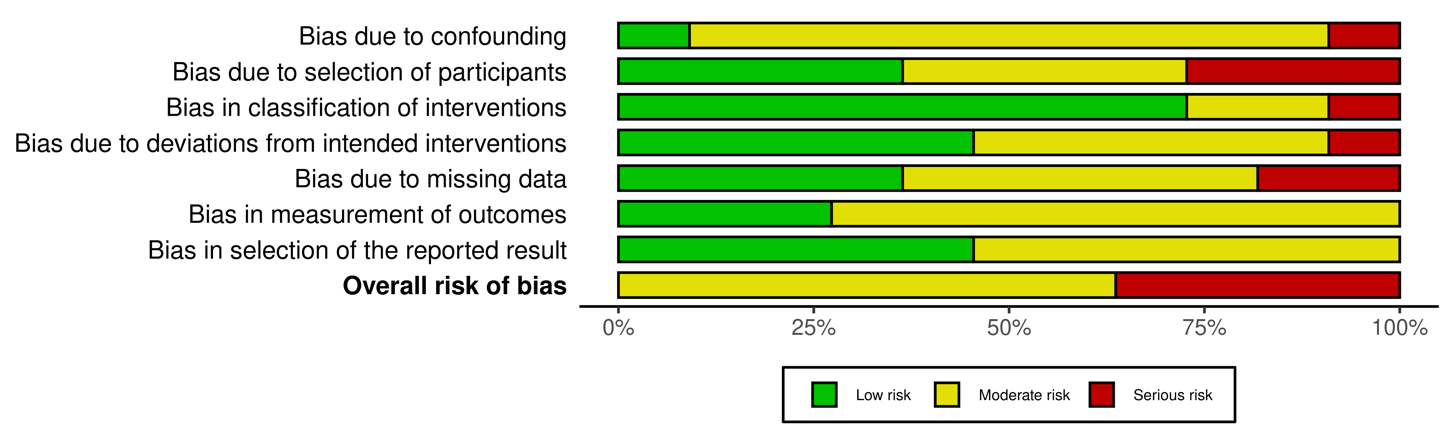
**

**b)**

**
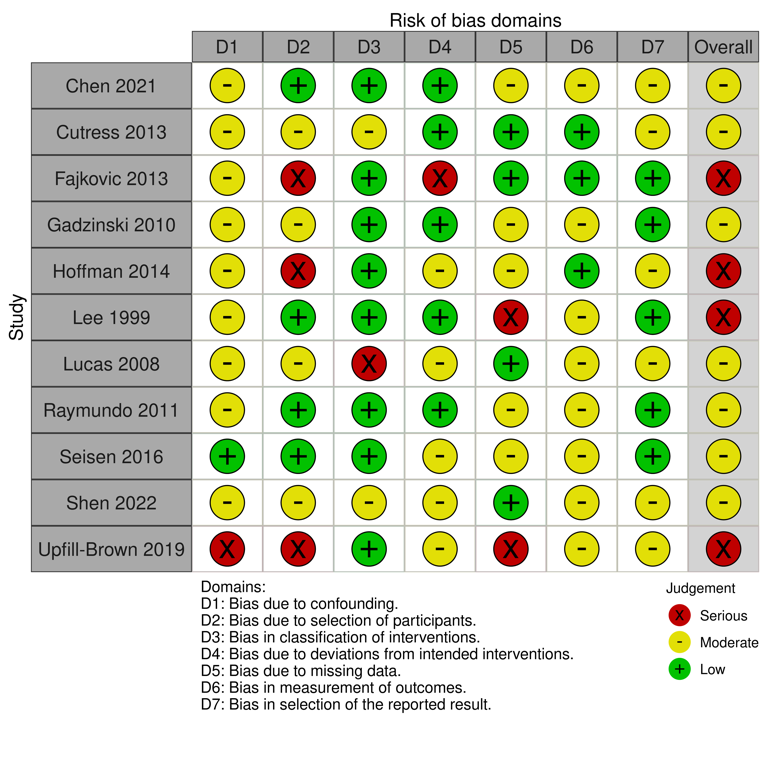
**
