# Supplementary material for: Endoscopic intervention versus radical nephroureterectomy for the management of localized upper urinary tract urothelial carcinoma: a systematic review and meta-analysis of comparative studies
Source: World J Urol. 2024 May 14;42(1):318. doi: 10.1007/s00345-024-05032-y (PMC11093876; doi:10.1007/s00345-024-05032-y)
Supplement: Supplementary file 2 — Supplementary file2 (DOCX 57 KB) [file 345_2024_5032_MOESM2_ESM.docx]

**Supplementary Figure 1:** *PRISMA 2009 flow diagram*

Studies from databases/registers **(n = 2614)**

Embase (n = 1453)

PubMed (n = 1095)

Scopus (n = 66)

References from other sources **(n = 0)**

**Identification**

Studies excluded **(n = 1851)**

Studies not retrieved **(n = 0)**

Studies assessed for eligibility **(n = 274)**

Studies sought for retrieval **(n = 274)**

Studies screened **(n = 2125)**

Duplicate references removed **(n = 489)**

Studies excluded **(n = 263)**

Review (n = 37)

Case report (n = 7)

Animal study (n = 1)

Wrong setting (n = 40)

Wrong outcomes (n = 1)

Not in English (n = 27)

Duplicated data (n = 6)

Meeting abstract (n = 103)

Letter to editor (n = 10)

Wrong intervention (n = 8)

Wrong study design (n = 8)

Low number of cases (n = 8)

Wrong patient population (n = 6)

No data for conservative managment (n = 1)

**Screening**

Studies included in review **(n = 11)**

**Included**
